# Mitochondrial Homeostasis Mediates Lipotoxicity in the Failing Myocardium

**DOI:** 10.3390/ijms22031498

**Published:** 2021-02-02

**Authors:** Tom Kretzschmar, Jasmine M. F. Wu, P. Christian Schulze

**Affiliations:** Department of Internal Medicine I, University Hospital Jena, 07747 Jena, Thüringen, Germany; tom.kretzschmar2@med.uni-jena.de (T.K.); jasmine.wu@med.uni-jena.de (J.M.F.W.)

**Keywords:** lipotoxicity, mitochondrial homeostasis, heart failure, metabolism

## Abstract

Heart failure remains the most common cause of death in the industrialized world. In spite of new therapeutic interventions that are constantly being developed, it is still not possible to completely protect against heart failure development and progression. This shows how much more research is necessary to understand the underlying mechanisms of this process. In this review, we give a detailed overview of the contribution of impaired mitochondrial dynamics and energy homeostasis during heart failure progression. In particular, we focus on the regulation of fatty acid metabolism and the effects of fatty acid accumulation on mitochondrial structural and functional homeostasis.

## 1. Introduction

Heart failure is manifested by the disability of the heart to maintain its pumping efficiency. In 2016, cardiovascular disease was responsible for 840,768 deaths in the US [1]. Therefore, it remains the most common cause of death in Western countries. The causes of heart failure are diverse; however, all can contribute to cardiomyocyte death and ultimately lead to cardiac dysfunction [1]. In addition to the symptoms typically observed in heart failure [2], metabolic alterations have been reported in heart failure patients [3,4,5]. Such changes have attracted increasing attention as novel biomarkers [6,7] as well as for drug targets [8,9]. Hage et al. recently identified different metabolic profiles between new-onset HFpEF and HFrEF patients [10]. Interestingly, Haase et al. demonstrated that the altered metabolic alterations in aortic stenosis patients could be partially reversed after transaortic valve replacement [11]. Among all the metabolites analyzed, fatty acids and fatty-acid-associated metabolites are predominantly affected. The changes in cardiac metabolism, especially the fatty acid β-oxidation, have always been a major research focus in the study of the development and progression of heart failure [12,13].

## 2. Metabolic Plasticity of the Developing Heart

The adult heart is a metabolic omnivore and relies mainly on fatty acid β-oxidation for energy production under physiological conditions [13]. This reliance is essential for the proliferation and maturation of cardiac myocytes, but it is not the primary energy source in early developmental stages [14]. During cardiac differentiation of pluripotent stem cells and early postnatal stages, glycolysis is utilized as the main source of ATP generation [15,16]. It was shown in 1991 that the glycolytic rate in a 1-day-old rabbit heart was higher than that in a 7-day-old heart, in which increased palmitate oxidation was observed [17]. Both in vitro [5,6] and in vivo [7] data demonstrate that there is a metabolic switch from cytoplasm-localized glycolysis to mitochondria-localized fatty acid β-oxidation. Some of the main regulators of such a switch are peroxisome proliferator-activated receptors (PPARs) [18,19]. PPARs belong to the class II nuclear hormone receptors and contain three isoforms: PPARα, PPARβ/δ, and PPARγ. They have diverse cellular functions and are involved in different metabolic and developmental signaling pathways [20,21,22]. PPARα is involved in transcriptional regulation of fatty acid β-oxidation related genes, such as carnitine palmitoyltransferase 1 (CPT1) [23], clusters of differentiation 36 (CD36) [24] and diacyl-glycerol acyltransferase (DGAT) [25]. Cao et al. showed that the metabolic switch from glycolysis to fatty acid β-oxidation in mice at an early post-natal stage is promoted by PPARα [14]. Despite being an important metabolic mediator in the early post-natal stage, the role of PPARα in the adult myocardium is more ambivalent. It has already been shown that PPARα ^−/−^ mice are characterized by reduced cardiac metabolism without significant effect on cardiac function at baseline level [26,27]. Additionally, Kalliora et al. demonstrated that the activation of PPARα/γ by the dual agonist tesaglitazar led to cardiotoxicity. This is mediated by the reduction and inhibition of cardiac PGC1α (PPARGC1A), a key regulator in mitochondrial metabolism and homeostasis [19]. The finding illustrates the diverse effects of PPARα during different developmental stages. Nevertheless, PPARα activation is directly linked to fatty acid β-oxidation and, therefore, mitochondrial metabolism. The maintenance of the mitochondria homeostasis is therefore crucial for not only cell vitality but also for the heart’s functional integrity.

## 3. Mitochondrial Metabolism and Mitochondria-Associated Disorders

Mitochondria are the main sites for ATP generation in all mammalian organisms. The electron transport chain (ETC) or respiratory chain converts inter alia NADH into ATP via its 5 subunits [28,29]. Briefly, Complex I is responsible for the removal of two electrons from NADH and transfers them to ubiquinone to create ubiquinol [30,31]. Complex II is also required for the generation of ubiquinol but from succinate instead of NADH [32]. Both complexes generate hydronium ions, which are pumped into the inner mitochondria membrane space to generate a proton gradient. Complex III provides the substrates for complex I and II by converting ubiquinol back to ubiquinone [33]. Complex III gets reduced during this process, and the hydronium ion gradient is further increased inside of the mitochondria. Complex IV oxidizes complex III and allows it to maintain its biological function [34,35,36]. Complex V uses all the generated hydronium ions to convert ADP to ATP. Complex V is an ion channel that transports the hydronium ion out of the mitochondria. During this process, complex V is moved in a rotational direction, which causes the oxidative phosphorylation of ADP to ATP [37,38]. Dysfunction in each respective complex leads to different diseases called mitochondrial respiratory chain disorders (MRCDs) [39,40]. The Kearns-Sayre syndrome [41], Pearson Syndrome [42], and Leigh syndrome [43] are MRCDs associated with the development of heart failure.

The necessary substrate i.e., NADH, is primarily provided by the tricarboxylic acid cycle (TCA cycle). Shortly, the TCA cycle is localized in the inner mitochondria matrix and converts initially supplied acetyl-CoA into different carboxylic acids. During these reactions, three NADH are generated inter alia, which are one of the basic substrates for the respiratory chain. While the TCA generates NADH mainly from acetyl-CoA, it is also possible to produce intermediate molecules like α-ketoglutarate from other sources e.g., glutamate [44]. Glutamate is an amino acid involved in various signaling pathways in a tissue-dependent manner [45]. In mitochondrial metabolism, glutamate is predominantly catalyzed by glutamate dehydrogenase (GDH) into α-ketoglutarate, which is then converted in the TCA to enable continuous NADH production [46]. Disruption of the glutamate metabolism is associated with diseases such as hyperinsulinism/hyperammonemia syndrome (HI/HA) [47], olivopontocerebellar degeneration [48], or retinal diseases [49].

One of the major ways to provide acetyl-CoA for the TCA is via pyruvate conversion. Pyruvate is the conjugated base of pyruvic acid and is catalyzed by pyruvate dehydrogenase to acetyl-CoA [50] or by pyruvate carboxylase to oxaloacetate [51]. Both molecules participate in the TCA cycle localized in the mitochondria matrix [52,53]. Pyruvate is initially produced at the end of glycolysis, and a dysregulated glycolytic pathway is associated with diseases such as hemolytic anemia [54].

The second way to provide acetyl-CoA for the TCA cycle is fatty acid oxidation, which serves as the most important mechanism in cardiac metabolism and is also known as β-oxidation [55]. In mitochondria, acetyl-CoA can also be generated via β-oxidation of acyl-CoA [56]. During fatty acid β-oxidation, free fatty acids are transported from the blood into the cells via fatty acid transport proteins (FATPs) e.g., CD36 [57,58,59]. The long chain fatty acid CoA ligase 1 (ACSL1) then converts the fatty acid under the utilization of CoA and ATP into acyl-CoA [60,61,62]. This intermediate molecule is attached to carnitine, creating acylcarnitine in the process, and is regulated by carnitine palmitoyltransferase I (CPTI) [63,64]. Afterwards, acylcarnitine is transported by carnitine–acylcarnitine translocase (CACT) [65] into the inner mitochondria matrix, where acyl-CoA is separated from the acylcarnitine by carnitine palmitoyltransferase II (CPTII) [66,67]. The initial carnitine is transported back into the cytoplasm again for the next acyl-CoA to bind. In the inner mitochondria matrix, acyl-CoA is further cleaved by continuous thiolysis [68], a process in which an acetyl-CoA molecule is split from the original acyl-CoA by 3-ketoacyl-CoA thiolase [69,70]. This process continues until the complete acyl-CoA is converted into acetyl-CoA. One thiolysis cycle generates five molecules of ATP, and thus making fatty acid β-oxidation the most efficient method for energy production.

An additional method of acetyl-CoA generation is via ketone body oxidation. Ketone bodies are metabolic products of fatty acid β-oxidation and are characterized by their keto group (C=O), with acetone as the simplest embodiment. They are primarily synthesized in the liver from acetyl-CoA when the plasma glucose level is low. The primarily circulating ketone body β-hydroxybutyrate (βOHB) is transported via the bloodstream into the heart. Inside the heart, monocarboxylate transporters (MCT1) transfer βOHB inside the mitochondria, where it is oxidized by βOHB dehydrogenase (BHD1) into acetoacetate. This intermediate molecule is further processed into acetyl-CoA for the TCA cycle [71]. Although ketone bodies do not contribute significantly to myocardial ATP synthesis, increased circulating levels of βOHB and ketone body uptake have been observed in heart failure patients [72,73,74,75,76]. However, Ho et al. demonstrated that increased ketone oxidation in hypertrophied hearts is not accompanied by improved cardiac efficiency [77]. Further research is necessary to understand if ketone bodies simply serve as an alternative energy source for the failing heart or if increased ketone body oxidation has long-term benefits [78,79,80].

The complexity and diversity of the involved molecules and signaling pathways show how regulation and function of mitochondria homeostasis are intertwined, especially the fatty acid metabolism for cardiac muscle cells [13,81]. The shift from physiological fatty acid β-oxidation of healthy mitochondria to lipotoxic fatty acid accumulation, which subsequently leads to mitochondrial damage and apoptosis, is associated with different cardiac dysfunctions [12]. Therefore, a better understanding of how mitochondrial fatty acid metabolism is impaired and its associated consequences are crucial for the development of new therapeutic interventions to treat heart failure patients.

## 4. Lipotoxicity and Mitochondrial Homeostasis

Numerous pathogenic factors are associated with the development and progression of heart failure, including accumulated adipose tissue and fatty acids. In recent years, the epicardial adipose tissue (EAT) has gained much attention due to its localization in the heart and versatile functions [82,83,84]. Under physiological conditions, the EAT fulfills cardioprotective functions e.g., by the secretion of anti-inflammatory and anti-atherosclerotic cytokines such as adiponectin and adrenomedullin [85,86]. EAT acts as an inflammatory mediator by recruiting activated macrophages, secreting proinflammatory cytokines, and increasing transport of fatty acids [87]. Although further research is necessary to determine the definitive role of EAT in heart failure development, it emphasizes the relevance of fatty acids in lipotoxic cardiomyopathy. Fatty acid accumulation, which arises from genetic disorders [88] or lifestyle [89], is associated with a chronic inflammatory state. The accumulation is a consequence of insufficient fatty acid β-oxidation caused by impaired mitochondrial function and occurs in advanced stages of heart failure. An elevated fatty acid level further promotes inflammation and mitochondrial damage. The inflammation is characterized by elevated production of inflammatory cytokines such as TNFα, IL-1ß, and IL-6 [90,91].

**Figure 1:**
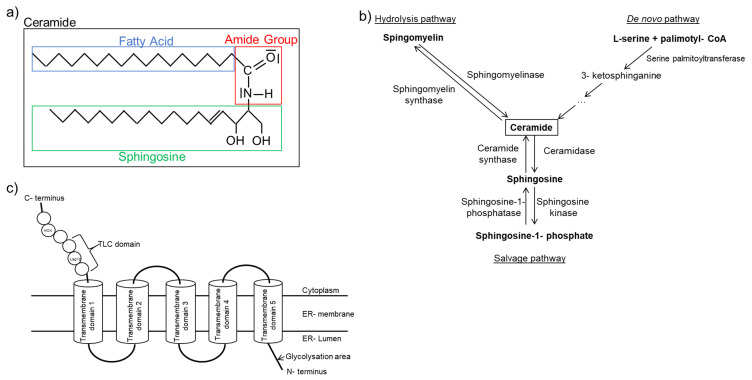
Ceramide structure and synthesis. (**a**) Schematic diagram illustrating ceramide structure. (**b**) Simplified ceramide synthesis pathways with essential enzymes in the respective pathway. (**c**) Schematic presentation of ceramide synthase structure based on previous findings [93,94,95,96,97,98,99,100,101].

One category of fatty acid species that has a strong association with inflammation is ceramide. Ceramides contain a sphingosine backbone with an attached fatty acid connected via the amide group. The attached fatty acid is of various length, and ceramides of different fatty acid length have diverse effects in cells (Figure 1a). Long chain (C > 14) and very long chain (C > 20) ceramides are increased in heart failure patients and are linked to a pro-inflammatory state [92].

Ceramides are synthesized via three separate pathways (Figure 1b). The *salvage* pathway is characterized by the constant catabolism of various complex sphingolipids by different enzymes in lysosomes [102]. The de novo pathway synthesizes ceramides from L-serine and palmitoyl-CoA. This enzymatic reaction is regulated by serine-palmitoyltransferase (SPT), which is the rate-limiting enzyme in this distinctive signaling pathway [103,104]. The generated ceramides differ in chain length based on the attached fatty acid. Knockout of serine-palmitoyltransferase long chain base subunit 2 (SPTLC2), one of the long chain subunits of SPT, is also responsible for its biological function, leading to dilated cardiomyopathy in older mice [105]. However, an improved cardiac function is observed after myocardial infarction due to reduced very long chain ceramides [106]. Furthermore, inhibition of SPT with myriocin [107] mitigates inflammation after ischemia/reperfusion following transient coronary occlusion in mice [108]. Myriocin treatment also improves mitochondrial function by increasing mRNA expression of metabolic related genes, including PPARα, PPARγ, CPT1a, and CPT1b after I/R, thus reducing tissue damage [109]. These findings imply that the SPT may serve as a potential target for heart failure treatment.

Ceramides can also be synthesized via direct attachment of a fatty acid to sphingosine. This reaction is catalyzed by one of six isoforms of ceramide synthase (CerS) [93] (Figure 1c). The human gene was first identified in 1991 and was initially labeled as upstream of growth and differentiation-1 [110]. Later, more isoforms were identified, and they were classified as ceramide synthases [96,98,111,112,113]. CerS displays tissue-dependent expression patterns, and different CerS have preference towards different fatty acid substrates [94]. For example, CerS2 shows ubiquitous expression in most tissues and synthesizes very long chain ceramides. It plays an important role as a tumor suppressor [114]. In recent years, CerS2 gained increased attention for its contribution to very long chain ceramide formation and cardiotoxicity. Law et al. showed that the overexpression of CerS2 led to increased very long chain ceramide accumulation and subsequently mitochondrial damage and autophagy in cardiomyocytes [115]. Similar effects were also observed for CerS5. Russo et al. demonstrated that lipotoxic cardiac hypertrophy caused by myristate (C14:0) is reduced in CerS5 knockdown cardiomyocytes, as characterized by the reduction of autophagy promoting genes [116]. CerS6, but not CerS5, is associated with mitochondrial fragmentation and the development of insulin resistance in obese mice [117]. Furthermore, knockout of CerS6 improves mitochondrial structure [118]. CerS3 and CerS4 are mostly associated with skin barriers and their function [119,120], while CerS1 is mostly involved in diseases associated with the nervous system [121]. Despite the origin of proinflammatory ceramides differing, the outcomes are the same. The lipotoxicity-associated damages in mitochondrial structure and function result in apoptosis and autophagy [122,123,124].

The mitochondrial structural integrity is essential for its function and viability. Under physiological conditions, the number of mitochondria differs between different cell types [125], and they display a broad net-like structure within the cell. The structural integrity is regulated by different mitochondrial fusion and fission proteins and there is a constant cycle between mitochondrial synthesis and degradation, also known as “mitochondrial dynamism”. Mitofusin 1 (MFN1) and Mitofusin 2 (MFN2) are two GTPases localized at the outer mitochondria membrane [126,127,128] which are responsible for outer membrane fusion as well as mediating the connection with endoplasmatic reticulum (ER) [129]. Owing to the important regulatory role in mitochondria structure, a problem with either MFN1 or MFN2 or both impairs physiological development [130]. Knockout of MFN1 in murine cardiomyocytes leads to an increased number of spherical mitochondria, while mitochondria function remains mostly intact [131]. Nevertheless, the translational inhibition of MFN1 via miR-140 promotes mitochondrial fission followed by apoptosis [132]. Furthermore, it has been shown that the interaction with β II protein kinase C (βIIPKC) leads to MFN1 inactivation and promotes mitochondrial degradation in rat cardiomyocytes during heart failure [133]. Homozygous MFN2 knockout is lethal due to severe disruption in the physiological cell layer of placental trophoblasts. Furthermore, the conditional knockout of both genes at the same time has also been shown to be lethal [130]. Specific deletion of MFN1 and MFN2 in adult cardiomyocytes caused mitochondrial fragmentation and subsequently dilated cardiomyopathy [134]. The knocking out of MFN2 in old cardiomyocytes alone only caused a mild form of cardiomyopathy but showed better recovery after reperfusion injury [135]. Interestingly, Hall et al. showed that the animals with both MFN1 and MFN2 knockout did not develop cardiomyopathy at the age of 8–10 weeks and were cardioprotective against acute myocardial infarction. Such protection was rendered by reduction of ROS due to reduced Ca^2+^ overload [136]. These results indicate that MFN1 and MFN2 seem to have age-dependent effects on mitochondrial function and cardiac vitality as ablation of both genes appears to have negligible impact in young animals. This implies that further research is necessary to better understand how mitochondrial structure is regulated other than by MFN1 and MFN2. Hu et al. recently showed that obese diabetic mice are characterized by reduced MFN2 expression and disrupted mitochondria. Reconstitution of depleted MFN2 with an adenoviral vector encoding MFN2 inhibited mitochondrial fission [137]. Additionally, transient knockdown of MFN2 in the murine skeletal muscle resulted in reduced Ca^2+^ uptake and reduced tether frequency [138].

Another protein involved in mitochondria structural integrity is dynamin-like 120 kDa protein, mitochondrial (OPA1). OPA1 is responsible for the inner membrane fusion and, therefore, cristae morphogenesis and function [139,140]. Next to optic atrophy, homozygous OPA1 mutation in humans is accompanied by hypertrophic cardiomyopathy [141]. Lipotoxicity generally causes mitochondrial damage, which is characterized by the downregulation of mitochondrial fusion-associated genes and the potential upregulation of fission-related genes to enhance mitochondrial degradation.

The center of mitochondrial fission is the dynamin-related protein 1 (DRP1), which belongs to the family of dynamins and is also a GTPase [142,143]. For the fission of mitochondria by DRP1, certain adaptor proteins and their interaction with the GTPase are necessary. Mitochondrial dynamics protein 49 (aka MIEF2) and 51 (aka MIEF1) are localized at the outer membrane of the mitochondria and recruit DRP1 [144,145]. Upon recruitment, DRP1 and MID49/51 encircle the mitochondria, which leads to the fission of the cell organelle via nucleotide-driven allostery [146,147,148,149]. Chen et al. showed that reduction of DRP1 by siDRP1 attenuated palmitate-induced apoptosis, therefore linking lipotoxicity to mitochondrial homeostasis [150]. It has been shown that DRP1 activity is affected by several post-translational modifications [151]. Kashatus et al. demonstrated that DRP1 phosphorylation by Erk2 led to mitochondrial fission [152]. Furthermore, a DRP1-mediated effect can be partially regulated by the outer mitochondria membrane-associated E3 ubiquitin ligase MARCH5 [153]. In addition, SUMOylation has also been identified as a post-translational modifier of DRP1 [154]. Lipotoxicity-induced ubiquitination could therefore affect DPR1 activity and promote mitochondrial fission [155]. Additional recruitment partners for DRP1 are mitochondrial fission factor (Mff) and fission, mitochondrial 1 (FIS1). Both Mff and FIS1 are also located at the outer mitochondria membrane and are able to recruit DRP1. However, Mff can also work independently of FIS1 [156]. While FIS1 is highly involved in mitochondrial degradation [157,158], it seems not to be indispensable for the general process of mitochondrial fission [156,159].

Disruption of mitochondrial structure is directly associated with mitochondrial function [160]. Zhao et al. demonstrated that the knockdown of MFN2 in Kunming white mice embryos led to reduced ATP content and increased apoptosis at the blastocyst stage, proving the importance of intact mitochondrial homeostasis during early embryonal development [161]. Furthermore, MFN2 ablation is associated with cardiac myopathy [113] and impaired insulin signaling in murine hepatic cells [162]. Furthermore, decreased glucose and fatty acid β-oxidation [163] and reduced mitochondrial ATP production are observed in skeletal muscle cells [164,165,166]. Reduction of mitochondrial fusion-related genes not only decreases mitochondrial function but also increases expression of fission-related genes. DRP1 is essential for healthy mitochondrial dynamism and cardiac knockout of DRP1 leads to mitochondria enlargement caused by impaired physiological mitophagy. This ultimately leads to cardiac cell death [167,168,169]. Nevertheless, it has been shown that DRP1 inhibition with mitochondrial division inhibitor-1 or P110 reduces in vitro cell death induced by simulated ischemia/reperfusion [141]. Furthermore, reduction in the myocardial infarct size has also been observed in vivo [170,171]. Similar results are observed for FIS1/DRP1 interaction during septic cardiomyopathy [172]. The authors showed that treatment of LPS induced septic mice with P110 reduced mortality rate, which was accompanied by improved mitochondrial structure and function. Furthermore, Hu et al. demonstrated that a high-fat diet led to increased DRP1 acetylation and the subsequent mitochondrial fission resulted in cardiotoxicity and cell death [173]. Although knocking out DRP1 is detrimental for embryonic development, its inhibition shortly after cardiomyopathy appears to be beneficial. These results show that the mitochondria structure is irreversibly connected to mitochondria function and that impairment of structural integrity leads to reduced ATP generation and cell death [174]. Collectively, these findings provide evidence showing that mitochondria homeostasis and vitality are not only involved in lipotoxicity-mediated heart failure but also in different forms of cardiomyopathies.

Mitochondrial homeostasis is directly linked to calcium (Ca^2+^) flux. Ca^2+^ signaling is essential for myocardial contractility, but Ca^2+^ overload is detrimental for cells. It has been shown that verapamil, a Ca^2+^ channel blocker, reduces Ca^2+^-induced cardiotoxicity and improves survival in Sprague Dawley rats [175]. Further studies have determined the role of Ca^2+^ level in heart failure [176,177,178,179], and an improved intracellular Ca^2+^ level has been proven to be beneficial for heart failure patients [180,181].

In addition to type 2 ryanodine receptor (RyR2) [182,183] and sarco/endoplasmic reticulum Ca^2+^ATPase (SERCA) [184,185], the B cell lymphoma 2 family (Bcl-2) has been identified as an important regulator of Ca^2+^ homeostasis and mitochondria-mediated apoptosis. The Bcl-2 family contains different proteins with different functions for apoptosis regulation [186]. Bcl-2 is localized in the ER and is actively transported to the outer mitochondrial membrane to inhibit apoptosis [187,188]. The Bcl-2 family members Bax and Bak promote apoptosis due to mitochondria membrane permeabilization and cytochrome C release [189]. The dual role of the Bcl-2 protein family in apoptosis has also been observed for Ca^2+^ homeostasis.

Ca^2+^ efflux from the ER is in part regulated by the inositol trisphosphate receptor (IP_3_R) [190]. Ca^2+^ uptake at the outer mitochondria membrane is regulated by voltage dependant anion channel (VDAC) [191]. It is then transposed to the inner membrane by mitochondrial calcium uniporter (MCU) [192]. The ER is connected to the outer mitochondria membrane through mitochondria-associated ER membranes (MAMs). MAMs are primarily connected via MFN2 and S100B [193,194] to enable Ca^2+^ transmission. It has been shown that different IP_3_R isoforms are located at MAMs and are in close proximity to VDAC. The juxtaposition facilitates Ca^2+^ transport from the ER into the outer mitochondrial membrane [195]. Subsequently, Ca^2+^ is transported into the inner mitochondrial membrane by MCU to activate the pyruvate dehydrogenase complex for ATP synthesis. Reduced Ca^2+^ transport into the mitochondria due to decreased IP_3_R-VDAC interaction is associated with mitochondrial dysfunction [196]. Interactions of Bcl-2 and Mcl-1 with all IP_3_R isoforms have been observed and are accompanied by increased Ca^2+^ release and reduced apoptosis [197]. Furthermore, Vander Heiden et al. demonstrated that recombinant Bcl-x(L) kept VDAC in an open configuration to maintain metabolic flux and prevent cytochrome c release [198].

The Bcl-2 family protein that plays an adverse role in Ca^2+^ transport is the BCL2/adenovirus E1B 19kDa protein-interacting protein 3 (BNIP3). BNIP3 is proapoptotic and induces autophagy. It has been shown that BNIP3 overexpression resulted in increased Ca^2+^ leakage from the ER and mitochondrial uptake. Mitochondrial Ca^2+^ accumulation leads to caspase-independent cell death due to the opening of the mitochondrial permeability transition pore (mPTP) [199,200]. Palmitate-overload-dependent lipotoxicity is associated with increased Ca^2+^ flux from the ER into the mitochondria and potentially involves BNIP3 [201]. Furthermore, BNIP3 contributes to mitochondria fragmentation and fission by the binding of OPA1, subsequently leading to Bax/Bak-dependent apoptosis [202,203,204]. BNIP3-mediated mitochondrial fragmentation is also associated with maladaptive mitochondrial autophagy (mitophagy) and cardiac hypertrophy [205,206,207].

Mitophagy is a form of macroautophagy, where defective proteins and cell organelles e.g., depolarized mitochondria, are phagocytized and subsequently degraded. The degraded products provide substrates for energy metabolism and prevent the accumulation of harmful proteins. Therefore, macrophagy plays a major role in the regulation of cell survival and shows protective as well as maladaptive effects in the cardiovascular system [208,209,210]. Ma et al. demonstrated that BNIP3 overexpression leads to autophagosome accumulation and reduced lysosomal clearance that subsequently results in cell death [211]. Furthermore, it is found that BNIP3-mediated mitophagy is regulated by oxidative stress in myocardial ischemia and reperfusion [212]. Additionally, Sebastián et al. showed that the lack of MFN2 in old mice induced BNIP3-mediated mitophagy in a ROS-dependent manner [213]. Increased ROS production, in part caused by proinflammatory ceramides, could therefore trigger the proapoptotic effects of BNIP3-induced mitophagy [214,215]. Further examinations are necessary to verify the proposed interconnection.

**Figure 2:**
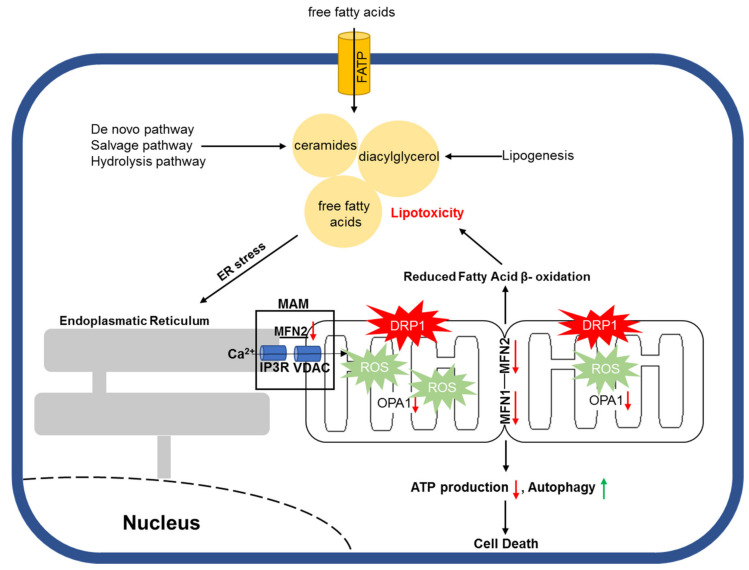
Schematic illustration of lipotoxicity-mediated mitochondrial damage.

One way by which accumulated toxic lipids impair ER and mitochondrial homeostasis is by increased reactive oxygen species (ROS). Under physiological conditions, ROS are important second messengers with a short half-life and are involved in various signaling pathways for cell survival [216,217,218]. However, excessive ROS production is negatively associated with cell vitality due to enhanced cellular damage and apoptosis [219,220] (Figure 2). Joseph et al. showed that NADPH oxidase 2 (NOX2) contributed to the formation of mitochondrial superoxides during LPS-mediated sepsis and that NOX2 inhibition with apocynin improved cardiac contractility in vivo [221]. In addition, Nakamura et al. proved that diabetic cardiomyopathy is associated with mitochondrial ROS accumulation as a result of increased p53/SCO2 activation following lipid accumulation in murine cardiomyocytes [222]. Furthermore, Tsushima et al. demonstrated that lipotoxicity caused by ACSL1 overexpression or palmitate or oleate treatment could lead to increased mitochondrial ROS production, which was accompanied by reduced mitochondrial ATP synthesis and enhanced mitochondrial fission in neonatal rat ventricular cardiomyocytes (NRVCs) [223]. Different lines of studies provide evidence that fatty acid accumulation is associated with increased mitochondrial ROS production, which is followed by impaired mitochondrial structure and function both in vitro and in vivo. Such an effect also appears to be cell type independent.

It is observed that lipid accumulation is a consequence of impaired fatty acid β-oxidation [13]. The cause of such impairment varies and includes obesity, diabetes, and cardiovascular disease [224,225,226]. However, Ji et al. demonstrated that SPTLC2 knockout mice show preserved cardiac function after myocardial infarction (MI) due to reduced proinflammatory ceramides [106]. Similar results were found by SPT inhibition with myriocin [109].

**Figure 3:**
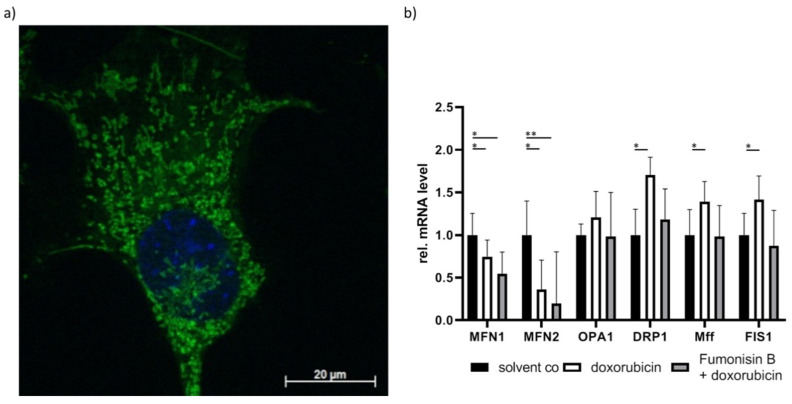
Visualization of mitochondrial structure. (**a**) Impaired and disturbed mitochondrial distribution was stained with MitoTracker (green) and visualized with laser scanning microscopy in doxorubicin-treated HL-1 murine cardiomyocytes. Nucleus was stained with Hoechst (blue). (**b**) mRNA expression of mitochondrial fusion and fission-related genes. Cardiac cell line HL-1 cells treated with doxorubicin showed decreased MFN1 and MFN2 expression but increased DRP1, Mff, and FIS1 expression. Doxorubicin-dependent induction of ceramide level was attenuated upon treatment with the unspecific CerS inhibitor fumonisin B. The fission-related mRNA expression (DRP1, Mff, FIS1) returned to non-significant levels following fumonisin B treatment. Significance was calculated with t-test. * *p* < 0.05, ** *p* < 0.01.

These results are also observed in our own research. We treated murine cardiomyocytes with doxorubicin and observed increased long chain and very long chain ceramides, which were accompanied by impaired mitochondrial distribution (Figure 3a). To determine if ceramides are a contributor to this effect, the cells were pre-treated with the unspecific CerS inhibitor fumonisin B. In addition to ceramide reduction, we also observed improved mitochondrial homeostasis (Figure 3b). To further investigate the role of ceramides in cardiotoxicity, CerS2-overexpressed cells were pre-treated with fumonisin B. We observed similar effects in CerS2-overexpressed cells. Therefore, ceramide accumulation could be an important contributor to doxorubicin-mediated cardiotoxicity (unpublished data). This indicates that lipotoxicity may play a causative role in cardiotoxicity. This also implicates that mitochondrial impairment arises from insufficient fatty acid β-oxidation, which then leads to fatty acid accumulation and promotes further mitochondrial damage. Further research is necessary to determine if cardiomyopathy is caused by toxic lipid accumulation, impaired fatty acid β-oxidation, or a combination of both.

## 5. Additional and Alternative Mitochondria Regulation

Mitochondria homeostasis is not affected by lipotoxicity alone. Other factors that influence myocardial metabolism also contribute to heart failure development. PKCδ has been found to lead to mitochondrial dysfunction by inducing ROS production in different disease models, including LPS-induced sepsis [227] and cardiac reperfusion [228,229]. Furthermore, it also promotes mitochondria-mediated apoptosis [230]. Sirtuin 1 (SIRT1), a histone deacetylase, is shown to be cardioprotective during I/R by activating the MAPK pathway and reducing ROS level [231]. Furthermore, it deacetylates and activates FoxO1 to promote cardioprotective autophagy during starvation [232]. Cardioprotective autophagy is reduced in diabetic cardiomyopathy [233]. SIRT1 positively regulates PGC-1α by maintaining and improving mitochondrial function [234]. SIRT3 is also involved in the maintenance of mitochondrial function [235] and is negatively regulated by miR-195 [236]. SIRT3 mitigates cardiac hypertrophy [237] and regulates acetylation and activation of 84 metabolism-associated proteins [238]. Krüppel-like factors (KLFs) belong to the family of zinc finger proteins and regulate the transcription of diverse genes. KLF4 is one of KLF isoforms that regulates physiological estrogen-related receptor/PPARγ coactivator 1 (ERR/PGC-1) functionality and is involved in the transcriptional regulation of autophagy [239]. KLF5 interacts with PPARα by directly binding on its promotor and is therefore directly involved in fatty acid β-oxidation [240]. On the other hand, KLF15 binds and activates the E1a-binding protein (p300) to regulate lipid flux [241]. In addition, the G protein-coupled receptor kinase 2 (GRK2) is negatively associated with cardiomyocyte survival after I/R by inducing mitochondrial dysfunction following translocation to the mitochondria [242,243].

## 6. Conclusions

**Figure 4:**
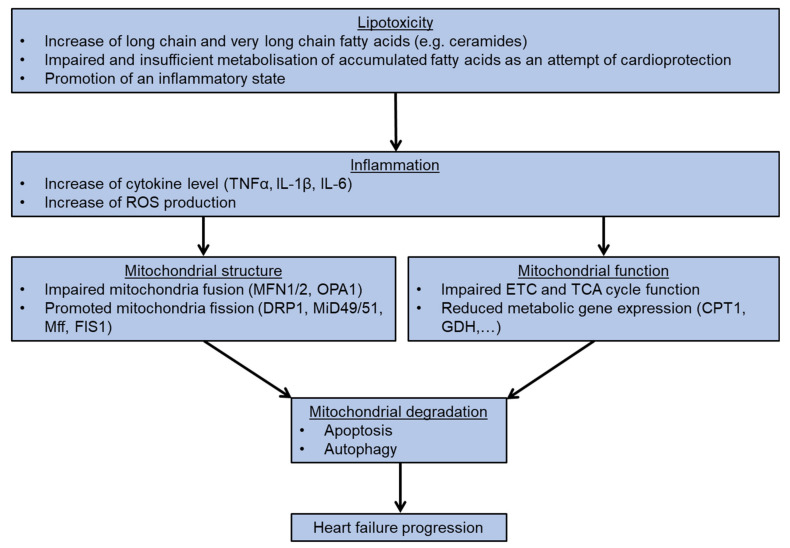
Flowchart presents the process of heart failure development following lipotoxicity.

In this review, we elaborate on the importance of mitochondria homeostasis in different forms of cardiomyopathies, in particular the effects of fatty acid accumulation and subsequent impairment of mitochondria structure and function. Both are essential for the development of cardiotoxicity and mitochondria-mediated cell death. The integrity of mitochondrial homeostasis is regulated by various factors, including the amount of long chain fatty acid levels e.g., ceramides, the mitochondrial ROS level, the status of mitochondrial fusion, and the fission and expression of metabolic genes. All these factors serve as valid and promising targets for the development of new therapeutic interventions for heart failure patients. (Figure 4).

## Data Availability

Data available in a publicly accessible repository.
